# Cerebellar transcranial magnetic stimulation for improving balance capacity and activity of daily living in stroke patients: a systematic review and meta-analysis

**DOI:** 10.1186/s12883-024-03720-1

**Published:** 2024-06-15

**Authors:** Jingfeng Wang, Zhisheng Wu, Shanshan Hong, Honghong Ye, Yi Zhang, Qiuxiang Lin, Zehuang Chen, Liling Zheng, Jiawei Qin

**Affiliations:** 1 Department of Rehabilitation Medicine, Quanzhou First Hospital Affiliated to Fujian Medical University, Quanzhou, China; 2 Department of Neurology, Quanzhou First Hospital Affiliated to Fujian Medical University, Quanzhou, China; 3 Department of Obstetrics and Gynecology, Quan Zhou Women’s and Children’s Hospital, Quanzhou, China; 4 Huada Street Community Health Service Center, Quanzhou, China; 5 Department of Cardiovascular Surgery, Quanzhou First Hospital Affiliated to Fujian Medical University, Quanzhou, China

**Keywords:** Cerebellum, Transcranial magnetic stimulation, Balance capacity, Activity of daily living, Systematic review, Meta-analysis

## Abstract

**Background:**

The application of cerebellar transcranial magnetic stimulation (TMS) in stroke patients has received increasing attention due to its neuromodulation mechanisms. However, studies on the effect and safety of cerebellar TMS to improve balance capacity and activity of daily living (ADL) for stroke patients are limited. This systematic review and meta-analysis aimed to investigate the effect and safety of cerebellar TMS on balance capacity and ADL in stroke patients.

**Method:**

A systematic search of seven electronic databases (PubMed, Embase, Web of Science, Cochrane Central Register of Controlled Trials, China National Knowledge Infrastructure, Wanfang and Chinese Scientific Journal) were conducted from their inception to October 20, 2023. The randomized controlled trials (RCTs) of cerebellar TMS on balance capacity and/or ADL in stroke patients were enrolled. The quality of included studies were assessed by Physiotherapy Evidence Database (PEDro) scale.

**Results:**

A total of 13 studies involving 542 participants were eligible. The pooled results from 8 studies with 357 participants showed that cerebellar TMS could significantly improve the post-intervention Berg balance scale (BBS) score (MD = 4.24, 95%CI = 2.19 to 6.29, *P* < 0.00001; heterogeneity, *I*^*2*^ = 74%, *P* = 0.0003). The pooled results from 4 studies with 173 participants showed that cerebellar TMS could significantly improve the post-intervention Time Up and Go (TUG) (MD=-1.51, 95%CI=-2.8 to -0.22, *P* = 0.02; heterogeneity, *I*^*2*^ = 0%, *P* = 0.41). The pooled results from 6 studies with 280 participants showed that cerebellar TMS could significantly improve the post-intervention ADL (MD = 7.75, 95%CI = 4.33 to 11.17, *P* < 0.00001; heterogeneity, *I*^*2*^ = 56%, *P* = 0.04). The subgroup analysis showed that cerebellar TMS could improve BBS post-intervention and ADL post-intervention for both subacute and chronic stage stroke patients. Cerebellar high frequency TMS could improve BBS post-intervention and ADL post-intervention. Cerebellar TMS could still improve BBS post-intervention and ADL post-intervention despite of different cerebellar TMS sessions (less and more than 10 TMS sessions), different total cerebellar TMS pulse per week (less and more than 4500 pulse/week), and different cerebellar TMS modes (repetitive TMS and Theta Burst Stimulation). None of the studies reported severe adverse events except mild side effects in three studies.

**Conclusions:**

Cerebellar TMS is an effective and safe technique for improving balance capacity and ADL in stroke patients. Further larger-sample, higher-quality, and longer follow-up RCTs are needed to explore the more reliable evidence of cerebellar TMS in the balance capacity and ADL, and clarify potential mechanisms.

**Supplementary Information:**

The online version contains supplementary material available at 10.1186/s12883-024-03720-1.

## Introduction

Stroke was the second most prevalent global cause of mortality and a foremost contributor to disability [1].Many stroke survivors still have functional disabilities despite prompt treatment, including balance dysfunction, decreased muscle strength, and sensory disorders [2]. These impairments, especially balance dysfunction, have substantial negative influence on functional independence and general recovery [3]. Balance dysfunction limits individuals’ capacity to engage in daily activities, markedly impacting their overall quality of life [2]. Rehabilitation program after stroke can improve patients’ functional impairments, however, the evidence of routine rehabilitation improving balance capacity and activity of daily living (ADL) were still conflicting [2]. Consequently, there is an imperative need for robust interventions focused on improving balance capacity and functional independence in stroke survivors.

Post-stroke, patients often experience a decrease in cortical excitability, functional disruption, vascular edema, and interhemispheric imbalance, which are crucial factors exacerbating their motor dysfunction [4]. In recent years, various rehabilitation technologies have rapidly developed. As an advanced non-invasive neuro-regulation technique, Transcranial Magnetic Stimulation (TMS) could promote stroke related motor function recovery by adjusting neuroplasticity [5]. TMS includes multiple modes, such as Repetitive Transcranial Magnetic Stimulation (rTMS), Theta Burst Stimulation (TBS), which further divides into Intermittent TBS (iTBS) and Continuous TBS (cTBS).

Contralesional low-frequency rTMS (LF_rTMS, ≤1HZ) and cTBS can inhibit, and ipsilesional high-frequency rTMS (HF_rTMS, >1HZ) and iTBS can promote the local cortical excitability [6–8].

TMS utilizes the principle of electromagnetic induction, and involves passing a current through a magnetic coil to generate a high-intensity, momentary magnetic field [9]. This field acts on the cerebral cortex to create an induced electric current within the brain, thereby affecting the membrane potential of neural cells [9]. Application of TMS in the early stages of stroke can reduce neuron death, increase neuron survival rates, and promote functional recovery [10, 11]. In the later stages of stroke, applying TMS to stimulate the cerebral cortex is aimed at recruiting or activating compensatory pathways and enhancing the adaptability and plasticity of the brain [10, 11].

Stimulation with TMS at primary motor cortex (M1) to improve motor function for stroke patients was common, and recognized by clinical practice [12]. While the M1 is a crucial structure involved in motor learning, the cerebellum is also one of the essential central regulators of bodily movement, participating in the regulation of body balance, muscle tension, and the coordination of voluntary movements [13–15]. This makes the cerebellum an attractive target for TMS in stroke rehabilitation, particularly given its interconnectedness with other key motor areas. Cerebellar stimulation could adjust M1 area, supplementary motor area, basal ganglia, and cingulate cortex, since the cerebellum is linked to these areas by nerve fibers [16]. Unlike traditional M1-focused TMS, cerebellar TMS offers a unique advantage by potentially influencing a broader network of motor-related regions, which could lead to more comprehensive improvements in motor function and balance. Cerebellar TMS may complement existing M1-focused treatments, providing a more holistic approach to stroke rehabilitation. Stimulation techniques targeting the cerebellum have started to gain attention in the recovery of motor functions after stroke. However, researches conducted to explore the effect of TMS for post-stroke balance function were still limited and small-sample.

To date, two reviews [17, 18] and one meta-analysis [19] have discussed the effect of cerebellar TMS on balance function and ADL for stroke patients. Ntakou et al. [17] and Xia et al. [18] just conducted narrative review without merging the data, so a comprehensive and realistic representation of the effect of cerebellar TMS on post-stroke balance function could be provided.

Moreover, both Ntakou et al. [17] and Xia et al. [18] only searched literature from English databases.

Additionally, Wu et al.’s meta-analysis retrieved relevant studies prior to October 2021, only including 4 studies for Berg Balance Scale (BBS) and 2 studies for ADL [19]. Over the past two years, many new studies have been published [20–22], updated evidence for cerebellar TMS treating post-stroke balance capacity and ADL are achieved. Therefore, the aim of this systematic review and meta-analysis is to integrate available good-quality RCTs and summarized the effect of cerebellar TMS on balance capacity and ADL in stroke survivors.

## Method

This systematic review and meta-analysis was conducted according to the Preferred Reporting Items for Systematic Reviews and Meta-Analysis (PRISMA) statement.

### Search strategy

A comprehensive literature search was performed in seven electronic databases, including PubMed, Embase, Cochrane Library (CENTRAL), Web of Science, China National Knowledge Infrastructure (CNKI), Wanfang and Chinese Scientific Journal (VIP) from their inception to October 20, 2023.

The following search items combined Medical Subject Headings and key words to identify appropriate studies: (“transcranial magnetic stimulation” or “magnetic stimulation transcranial” or “stimulation transcranial magnetic” or “theta burst stimulation” or “iTBS” or “cTBS” or “TMS” or “rTMS”) AND (“Stroke” or “cerebrovascular accident” or “CVA” or “cerebrovascular apoplexy” or “brain vascular accident” or “cerebrovascular stroke” or “cerebral stroke” or “cerebrovascular accident”) AND (“cerebellum” or “corpus cerebelli” or “cerebellar” or “parencephalon”).The full search strategy in PubMed database was available in Supplementary Table 1. The reference of all included studies were manually screened to identify any missed eligible study. Endnote X9 (Thomson Reuters) was used to manage all references.

### Inclusion and exclusion criteria

Inclusion criteria were: [1] target population: stroke survivor; [2] interventions: any type of transcranial magnetic stimulation on cerebellum, including single-pulse TMS, rTMS, cTBS, and iTBS; [3]comparisons: TMS vs. sham TMS/waitlist/non-treatment, TMS + conventional rehabilitation program vs. sham TMS + conventional rehabilitation program, TMS + conventional rehabilitation program vs. conventional rehabilitation program; [4]outcomes: at least one of balance capacity and activity of daily life measurements, such as Berg Balance Scale (BBS), TUGT (Time Up and Go Test), 10 m Walking Test (10MWT), Postural sway parameters, Stability Index (SI), Barthel Index (BI) and Modified Barthel Index (MBI); [5] study design: randomized controlled trials. Exclusion criteria were: [1] animal model; [2] repeated publications; [3] case reports, review, protocol, conference abstract, and letters to editor; [4] did not report balance capacity or ADL related outcomes.

### Data extraction

Firstly, duplicated references were removed by Endnote X9, two reviewers screened the titles and abstracts of including literatures to exclude obvious irrelevant references. Then, the reviewers carefully browse the full-text to identify the eligible studies. Finally, the relevant information were extracted to a pre-specified study characteristic form, including study author, study publication years, information of participants (numbers, age, gender, and stroke duration), type of stroke, intervention details, outcomes assessments, and adverse events. Any discrepancies were resolved by a third reviewer.

### Risk of bias assessment

The Physiotherapy Evidence Database (PEDro) scale was used to assess the included literatures’ methodological quality by two reviewers. The PEDro scale contained 11 items, including random allocation; concealed allocation; baseline comparability; blinding subjects; blinding therapists; blinding assessors; adequate follow-up; intention-to-treat analysis; between group comparisons; point estimates; and variability. Each item was assessed by “1” (satisfied) or “0” (not satisfied) with a maximum score of 10 points. 6 or higher points were categorized as high quality, 4–5 points were categorized as moderate quality, less than 4 points were categorized as poor quality. Any disagreements were discussed and resolved by a third reviewer.

### Data synthesis and analysis

Meta-analysis was conducted by Review Manager (RevMan 5.3, The Nordic Cochrane Center, The Cochrane Collaboration, Copenhagen, Denmark) software. The effect size were calculated by mean difference (MD) with 95% confidence interval (CI). The mean differences and standard deviations (SD) were extracted from baseline to post-intervention or follow-up for both experimental and control groups in each study. The net changes of outcomes assessment were more sensitive and appropriate to examine the pre-post difference in the interventional trial. For those studies only report the baseline, post-intervention and/or follow-up values, the means and SDs of the changes were calculated according to the method in the Cochrane Handbook for Systematic Reviews of Interventions [23]. For those studies only report means and 95%CI, the SD was calculated by dividing the 95%CI length by 3.92, then multiplied by the square root of sample size. If the change score SD was not reported, it would be calculated by the following formula,

$$\sqrt{{\text{S}\text{D}\text{b}\text{a}\text{s}\text{e}\text{l}\text{i}\text{n}\text{e}}^{2}+{\text{S}\text{D}\text{f}\text{i}\text{n}\text{a}\text{l}}^{2}-(2\times \text{C}\text{o}\text{r}\text{r}\times \text{S}\text{D}\text{b}\text{a}\text{s}\text{e}\text{l}\text{i}\text{n}\text{e}\times \text{S}\text{D}\text{f}\text{i}\text{n}\text{a}\text{l})},$$

and the correlation value was assigned as 0.5 [23]. Meta-analysis was performed separately for different outcomes. Heterogeneity was evaluated by Chi-square test and qualified by I^2^ statistic, interpreted as low ≤ 25%, moderate > 25% and ≤ 75%, or high>75. Random effect model was adopted for all meta-analysis if heterogeneity among studies was high. The data were extracted by the assistance with GetData Graph Digitizer 2.25 (http://getdata-grpah-digitizer.com/) if the original data was presented by graphs in the studies.

Sensitivity test was conducted to check the stability of the pooled results, and explore the possible heterogeneity resource. Subgroup analysis was conducted based on stroke stage (subacute, chronic), TMS protocol frequency (high-frequency, low-frequency), TMS type (rTMS, TBS), total TMS sessions (≤ 10 sessions, > 10 sessions), and TMS pulse per week (≤ 4500 pulse/week, > 4500 pulse/week). Funnel plot asymmetry test was conducted to determine the publication bias when a meta-analysis contain at least 10 studies.

## Results

### Study selection

**Fig. 1:**
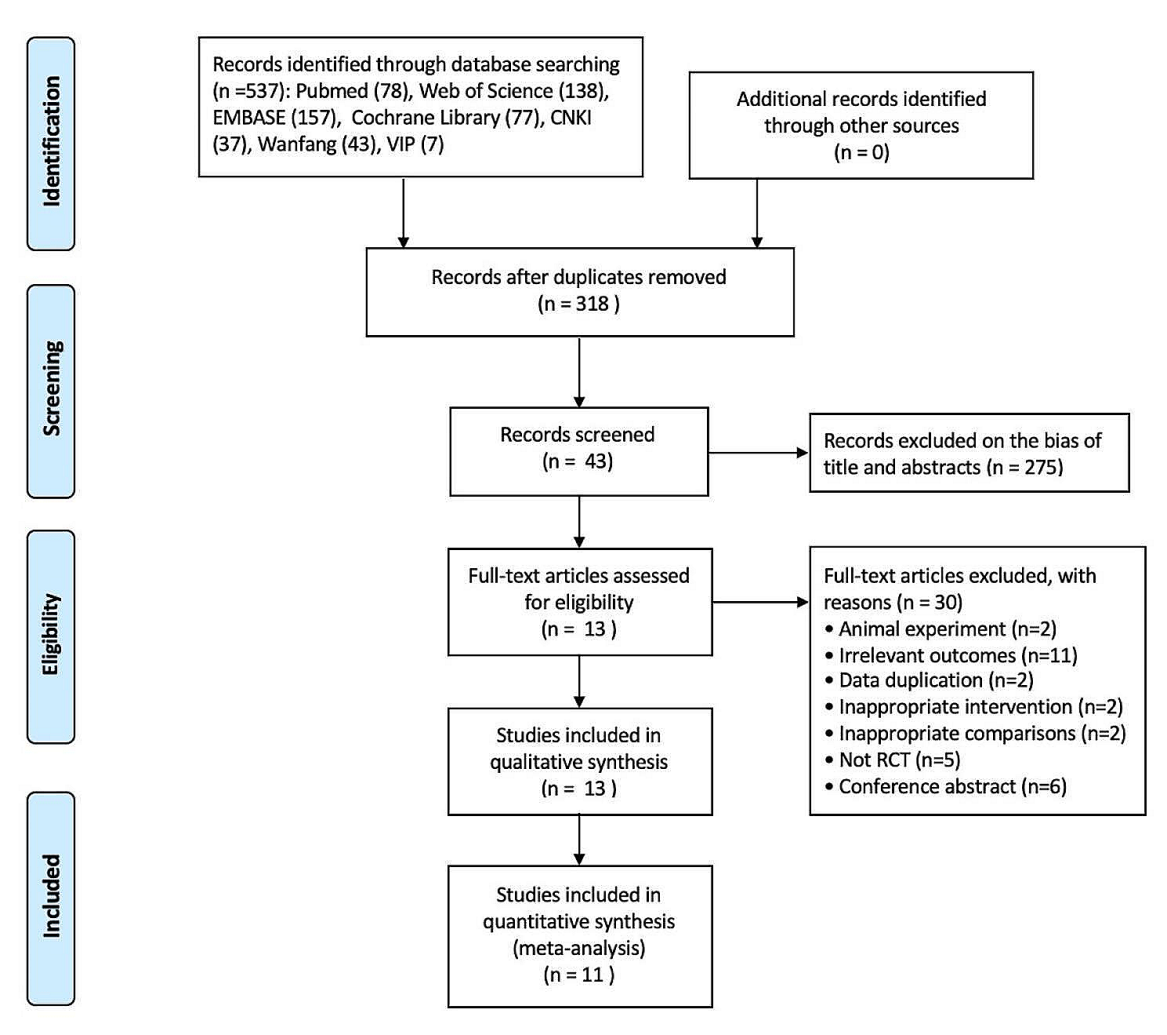
Flow diagram for studies selection

The flow diagram of literature search and study selection was shown as Fig. 1. A total of 537 articles from 7 electronic databases were retrieved. 13 RCTs with 542 participants were finally included in the systematic review and meta-analysis after removing duplicates, screening the titles and abstracts, and assessing the full-text of relevant literature.

### Characteristics of included studies

Of the 13 included RCTs, 8 articles were written in English [20–22, 24–28], 5 were written in Chinese [29–33]. The demographic and clinical characteristic of included studies were presented in Table 1.

The sample size of the included studies ranged from 27 to 82. The mean age of participants was between 46.8 and 75.9 years old. There were more male participants than female in the most included studies. 9 out of 13 included studies consisted of participants with subacute stroke, 3 out of 13 included studies consisted of participants with chronic stroke, only 1 study did not report the stroke stage of participants. Most of the participants were with the onset of ischemic stroke, only 1 study did not report the stroke type of the participants.

**Table 1:** Characteristics of including studies

Study (author /year)	No. of participant	Participant age (Mean ± SD, year)	Gender (F/M)	Course of stroke	Type of stroke (I/H)	Intervention	Cerebellar TMS protocol	Outcome measure	AE
Xie et al. 2021	EG: 18	EG: 52.35 ± 8.62	EG: 5/13	EG: 2.22 ± 1.7 m	EG: 10/8	EG: iTBS + PT	contralesional cerebellum iTBS, 80% AMT, 600 pulses/session, 1 session/day, 5 sessions/week, 2 weeks.	10 MWT (Comfortable Walking Time, Maximum Walking Time), TUG	NR
	CG: 18	CG: 54.41 ± 7.01	CG: 7/11	CG: 2.91 ± 1.96 m	CG: 10/8	CG: sham iTBS + PT			
Liao et al. 2021	EG: 15	EG: 51.53 ± 9.22	EG: 3/12	EG: 70.40 ± 44.43 d	EG: 7/8	EG: iTBS + PT	contralesional cerebellum iTBS, 80% AMT, 600 pulses/session, 1 session/day, 5 sessions/week, 2 weeks.	BBS, BI	Yes
	CG: 15	CG: 55.40 ± 8.10	CG: 6/9	CG: 86.53 ± 45.26 d	CG: 8/7	CG: sham iTBS + PT			
Li et al. 2021	EG: 30	EG: 56.77 ± 8.58	EG: 10/20	EG: 3.63 ± 1.85 m	EG: 24/6	EG: cTBS + M1 LF_rTMS + PT + OT + acupuncture	ipsilesional cerebellum cTBS, 80% AMT, 1200 pulses/session, 1 session/day, 6 sessions/week, 4 weeks.	MBI	NR
	CG: 30	CG: 57.60 ± 7.40	CG: 11/19	CG: 3.80 ± 1.71 m	CG: 23/7	CG: M1 LF-rTMS + PT + OT + acupuncture			
Koch et al. 2018	EG: 17	EG: 62.06 ± 12.05	EG: 4/13	EG: 14.11 ± 17.3 m	EG: 17/0	EG: iTBS + PT	contralesional cerebellum iTBS, 80% AMT, 1200 pulses/session, 1 session/day, 5 sessions/week, 3 weeks.	BBS, BI	NR
	CG:17	CG: 66.5 ± 10.35	CG:7/10	CG: 11.88 ± 17.56 m	CG: 17/0	CG: sham iTBS + PT			
Kim et al. 2014	EG: 22	EG: 67.4 ± 7.8	EG: 11/11	EG: 16.2 ± 13 d	/	EG: rTMS + PT	ipsilesional cerebellum rTMS, 100% RMT, 1 HZ, 900 pulses/session, 1 session/day for 5 days	10MWT (Comfortable Walking Time, Steps), BBS	NR
	CG:10	CG: 64.8 ± 11.7	CG: 4/6	CG: 15.1 ± 5.1 d	/	CG: sham rTMS + PT			
Im et al. 2022	EG: 15	EG: 75.13 ± 2.75	EG: 0/15	EG: 35.67 ± 43.27 m	EG: 15/0	EG: rTMS + PT	ipsilesional cerebellum rTMS, 90% RMT, 1 HZ, 900 pulses/session, 1 session/day, 5 sessions/week, 2 weeks	BBS, TUG, 10MWT(Comfortable Walking Time), ABC	Yes
	CG:16	CG: 75.94 ± 4.57	CG: 1/15	CG: 35.75 ± 45.12 m	CG: 16/0	CG: sham rTMS + PT			
Chen et al. 2021	EG: 16	EG: 57.38 ± 8.04	EG: 3/13	EG: 80.13 ± 35.19 d	EG: 10/6	EG: iTBS + PT	contralesional cerebellum iTBS, 80% AMT, 600 pulses/session, 1 session/day, 5 sessions/week, 2 weeks.	BI	NR
	CG: 16	CG: 51.44 ± 9.19	CG: 4/12	CG: 101.50 ± 54.15 d	CG: 8/8	CG: sham iTBS + PT			
Cha 2017	EG: 15	EG: 61.60 ± 7.76	EG: 7/8	EG: 75.20 ± 12.91 d	EG: 10/5	EG: rTMS + mirror therapy	ipsilesional cerebellum rTMS, 100% RMT, 1 HZ, 900 pulses/session, 1 session/day, 5 sessions/week, 4 weeks	TUG, Postural sway by Gaitview System	NR
	CG: 15	CG: 63.73 ± 6.10	CG: 8/7	CG: 77.20 ± 10.02 d	CG: 9/6	CG: sham rTMS + mirror therapy			
Wang et al. 2022	EG: 21	EG: 52.62 ± 8.61	EG: 10/11	EG: 82.33 ± 45.27 w	EG: 12/9	EG: iTBS + PT + OT	contralesional cerebellum iTBS, 80% AMT, 600 pulses/session, 1 session/day, 5 sessions/week, 4 weeks.	BBS, MBI	NR
	CG: 21	CG:54.62 ± 7.85	CG: 12/9	CG: 72.95 ± 47.37 w	CG: 13/8	CG: PT + OT			
Zhang et al. 2019	EG: 15	EG: 54.33 ± 11.46	EG:5/10	EG: 5.20 ± 3.60 w	EG: 9/6	EG: rTMS + PT + OT	contralesional cerebellum rTMS, 80% RMT, 10 HZ, 1200 pulses/session, 1 session/day, 5 sessions/week, 2 weeks.	BBS, SI by Tetrax Balance System	NR
	CG: 15	CG: 55.53 ± 13.13	CG: 6/9	CG: 5.47 ± 2.58 w	CG: 8/7	CG: sham rTMS + PT + OT			
Mao et al. 2021	EG: 41	EG: 59.12 ± 1.28	EG: 19/22	/	EG: 24/17	EG: rTMS + PT + OT	contralesional cerebellum rTMS, 110% RMT, 10 HZ, 1200 pulses/session, 1 session/day, 6 sessions/week, 3 weeks.	BBS, SI by Tetrax Balance System	NR
	CG: 41	CG: 59.33 ± 1.54	CG: 18/23	/	CG: 23/18	CG: sham rTMS + PT + OT			
Ding et al. 2022	EG: 38	EG: 56.65 ± 9.54	EG: 12/26	EG: 5.43 ± 1.54 w	EG: 38/0	EG: rTMS + PT	contralesional cerebellum rTMS, 80% RMT, 10 HZ, 1200 pulses/session, 1 session/day, 7 sessions/week, 3 weeks.	TUG, BBS, SI by B-PHY Balance System	NR
	CG: 38	CG:56.31 ± 9.14	CG: 14/24	CG: 5.37 ± 1.78 w	CG: 38/0	CG: sham rTMS + PT			
Duan et al. 2020	EG: 13	EG: 46.77 ± 9.56	EG: 6/7	EG: 5.77 ± 1.24 w	EG: 13/0	EG: rTMS + PT	ipsilesional cerebellum rTMS, 80% RMT, 1 HZ, 1600 pulses/session, 1 session/day, 7 sessions/week, 4 weeks	FMA-Balance	Yes
	CG: 14	CG: 47.86 ± 6.50	CG: 8/6	CG: 4.93 ± 1.00 w	CG: 14/0	CG: sham rTMS + PT			

Note: EG = Experimental Group; CG = Control Group; SD = Standard Deviation; F/M = Femal/Male; I/H = Ischemic/hemorrhagic; TMS = Transcranial Magnetic Stimulation; m = month; w = week; d = day; iTBS = intermittent Theta Burst Stimulation; cTBS = continuous Theta Burst Stimulation; rTMS = repetitive Transcranial Magnetic Stimulation; M1 = primary motor cortex; LF = Low Frequency; PT = Physical Therapy; OT = Occupational Therapy; AMT = Active Motor Threshold; RMT = Resting Motor Threshold; 10MWT = 10 m Walking Test; TUG = Time Up and Go; BBS = Berg Balance Scale; BI = Barthel Index; MBI = Modified Barthel Index; ABC = Activity-specific Balance Confidence; SI = Stability Index; FMA = Fugl-Meyer Assessment; AE = Adverse Event; NR = Not Reported

**Table 2:** Overall and subgroup analysis

Outcomes	Overall and subgroup analysis	No. of study	No. of participant	MD (95% CI)	*P*-value	Heterogeneity		
						Chi^2^	*P*-value	I^2^
BBS post-intervention	overall	8	357	4.24 (2.19, 6.29)	< 0.00001	27.07	0.0003	74%
	subacute	4	168	4.34 (2.68, 6)	< 0.00001	3.07	0.38	2%
	chronic	3	107	3.28 (0.22, 6.35)	< 0.00001	5.8	0.06	65%
	HF_TMS	6	294	4.42 (2.19, 6.64)	0.0001	26.66	< 0.0001	81%
	LF_TMS	2	63	2.09 (-4.44, 8.61)	0.53	0.03	0.86	0%
	≤ 10 sessions	4	123	2.98 (0.69, 5.28)	0.01	0.92	0.82	0%
	> 10 sessions	4	234	5.02 (2.1, 7.94)	0.0007	25.01	< 0.0001	88%
	≤ 4500 pulse/week	4	135	1.71 (0.37, 3.05)	0.01	0.05	1	0%
	> 4500 pulse/week	4	222	6.05 (4.86, 7.24)	< 0.00001	4.51	0.21	33%
	rTMS	5	251	5.67 (3.90, 7.44)	< 0.00001	5.85	0.21	32%
	TBS	3	106	2.95 (0.29, 5.61)	0.03	5.98	0.05	67%
BBS follow-up	overall	3	97	6.29 (3.81, 8.77)	< 0.00001	1.75	0.42	0%
	subacute	2	63	2.36 (-3.98, 8.7)	0.47	0.01	0.94	0%
	chronic	1	34	7 (4.3, 9.7)	< 0.00001	/	/	/
	HF_TMS	1	34	7 (4.3, 9.7)	< 0.00001	/	/	/
	LF_TMS	2	63	2.36 (-3.98, 8.7)	0.47	0.01	0.94	0%
	≤ 10 sessions	2	63	2.36 (-3.98, 8.7)	0.47	0.01	0.94	0%
	> 10 sessions	1	34	7 (4.3, 9.7)	< 0.00001	/	/	/
	≤ 4500 pulse/week	2	63	2.36 (-3.98, 8.7)	0.47	0.01	0.94	0%
	> 4500 pulse/week	1	34	7 (4.3, 9.7)	< 0.00001	/	/	/
	rTMS	2	63	2.36 (-3.98, 8.7)	0.47	0.01	0.94	0%
	TBS	1	34	7 (4.3, 9.7)	< 0.00001	/	/	/
TUG post-intervention	overall	4	173	-1.51 (-2.8, -0.22)	0.02	2.85	0.41	0%
	subacute	3	142	-1.47 (-2.77, -0.17)	0.03	2.59	0.27	23%
	chronic	1	31	-4.37 (-25.33, 6.59)	0.43	/	/	/
	HF_TMS	2	112	-0.93 (-2.4, 0.53)	0.21	0.16	0.69	0%
	LF_TMS	2	61	-3.52 (-6.24, -0.79)	0.01	0.02	0.87	0%
	≤ 10 sessions	2	67	-1.76 (-9.95, 6.43)	0.67	0.49	0.48	0%
	> 10 sessions	2	106	-1.5 (-2.81, -0.2)	0.02	2.36	0.12	58%
	≤ 4500 pulse/week	3	97	-3.28 (-5.94, -0.62)	0.02	0.64	0.73	0%
	> 4500 pulse/week	1	76	-0.97 (-2.44, 0.5)	0.2	/	/	/
	rTMS	3	137	-1.54 (-2.84, -0.25)	0.02	2.62	0.27	24%
	TBS	1	36	1.53 (-10.78, 13.84)	0.81	/	/	/
	PEDro score ≥ 6	3	143	-0.99 (-2.44, 0.45)	0.18	0.53	0.77	0%
	PEDro score < 6	1	30	-3.46 (-6.28, -0.64)	0.02	/	/	/
TUG follow-up	overall	1	31	-4.25 (-15.18, 6.68)	0.45	/	/	/
ADL post-intervention	overall	6	280	7.75 (4.33, 11.17)	< 0.00001	11.44	0.04	56%
	subacute	3	122	8.53 (6.19, 10.86)	< 0.00001	1.89	0.39	0%
	chronic	2	76	5.29 (1.59, 9)	0.005	1.42	0.23	29%
	HF_TMS	5	220	7.95 (4.15, 11.75)	< 0.00001	10.99	0.03	64%
	LF_TMS	1	60	5.36 (-3.09, 14.62)	0.26	/	/	/
	≤ 10 sessions	2	62	7.78 (2.4, 13.15)	0.005	1.41	0.23	29%
	> 10 sessions	4	218	7.68 (2.18, 13.18)	0.006	9.89	0.02	70%
	≤ 4500 pulse/week	3	104	5.71 (0.32, 11.1)	0.04	5.07	0.08	61%
	> 4500 pulse/week	3	176	9.54 (3.66, 15.42)	0.001	5.49	0.06	64%
	rTMS	1	82	14.81 (9.41, 20.21)	< 0.00001	/	/	/
	TBS	5	198	7.6 (5.63, 9.58)	< 0.00001	5.4	0.25	26%
ADL follow-up	overall	1	34	1.69 (-2.85, 6.23))	0.47	/	/	/
10MWT (comfortable walking time) post-intervention	overall	3	99	-3.25 (-9.76, 3.27)	0.33	0.18	0.92	0%
	subacute	2	68	-3.38 (-10.11, 3.34)	0.32	0.15	0.7	0%
	chronic	1	31	-1.11 (-27.53, 25.31)	0.93	/	/	/
	HF_TMS	1	36	-3.61 (-10.43, 3.21)	0.3	/	/	/
	LF_TMS	2	63	0.56 (-21.49, 22.62)	0.96	0.05	0.82	0%
10MWT (comfortable walking time) follow-up	overall	2	63	-0.31 (-22.06, 21.43)	0.98	0.066	0.8	0%
	subacute	1	32	3.6(-33.58, 40.78)	0.85	/	/	/
	chronic	1	31	-2.35 (-29.16, 24.46)	0.86	/	/	/
10MWT (steps) post-intervention	overall	1	32	-2 (-13.84,9.84)	0.74	/	/	/
10MWT (steps) follow-up	overall	1	32	-4.2 (-16.09, 7.69)	0.49	/	/	/
10MWT (maximum walking time) post-intervention	overall	1	36	-1.91 (-7.67, 3.85)	0.52	/	/	/
ABC post-intervention	overall	1	31	7.94 (-6.86, 22.74)	0.29	/	/	/
ABC follow-up	overall	1	31	8.31 (-6.26, 22.88)	0.26	/	/	/
SI (eyes open) post-intervention	overall	2	112	-4.47 (-6.19, -2.76)	< 0.00001	0.07	0.79	0%
SI (eyes close) post-intervention	overall	3	188	-4.37 (-5.58, -3.15)	< 0.00001	0.87	0.65	0%
	≤ 10 sessions	1	30	-3.86 (-6.85, -0.87)	0.01	/	/	/
	> 10 sessions	2	158	-4.47 (-5.8, -3.14)	< 0.00001	0.74	0.39	0%
postural sway (mm) post-intervention	overall	1	30	-10.06 (-21.19, 1.07)	0.08	/	/	/
FMA-Balance post-intervention	overall	1	27	1.53 (0.68, 2.38)	0.0004	/	/	/

Note: BBS = Berg Balance Scale; TUG = Time Up and Go; ADL = Activity of Daily Living; 10MWT = 10 m Walking Test; ABC = Activity-specific Balance Confidence; SI = Stability Index; FMA = Fugl-Meyer Assessment; HF = High Frequency; LF = Low Frequency; TMS = Transcranial Magnetic Stimulation; rTMS = repetitive Transcranial Magnetic Stimulation; TBS = Theta Burst Stimulation; MD = Mean Difference; CI = Confidence Interval

The intervention of experimental group were TMS combined with other rehabilitation program (Physical therapy, Occupational therapy, acupuncture et al.) in 11 included studies, sham TMS combined with the same rehabilitation program were in the control groups. 2 studies reported the additional TMS effects based on the conventional rehabilitation programs. About the TMS protocols, 5 studies used cerebellar iTBS, 1 study used cerebellar cTBS, and the other 7 studies used cerebellar rTMS (3 studies used high-frequency rTMS with 10HZ, and 4 studies used low-frequency rTMS with 1 HZ ). Regarding the stimulating sites of TMS, the ipsilesional cerebellum was targeted in the high-frequency TMS protocols, and the contralesional cerebellum was targeted in the low-frequency TMS protocols. The TMS protocols range from 5 days to 4 weeks with 5–28 sessions.

After reviewing all relevant outcome data of included studies, 1 study’s data [22] could be used directly, 10 studies’ data [21, 24, 25, 27–33] were calculated by the formula in the data synthesis and analysis section, 2 studies’ data [20, 26] were obtained through GetData software.10 studies only assessed the post-intervention effects of cerebellar TMS on balance capacity and/or ADL. 3 studies assessed both the post-intervention and follow-up effects of cerebellar TMS on balance capacity and/or ADL. The outcomes of balance ability were the clinical and experimental tests, including Berg Balance Scale (BBS), Time Up and Go (TUG), 10 m Walking Test (10MWT), Stability Index (SI) and postural sway parameters assessed by specific equipment, Activity-Specific Balance Confidence (ABC) scale, and Fugl-Meyer Assessment Balance (FMA-Balance). The 10MWT assessment parameters contained comfortable walking time, maximum walking time, test completion steps. The outcomes of ADL were Barthel Index (BI) and Modified Barthel Index (MBI).

### Effects of cerebellar TMS on BBS score

**Fig. 2:**
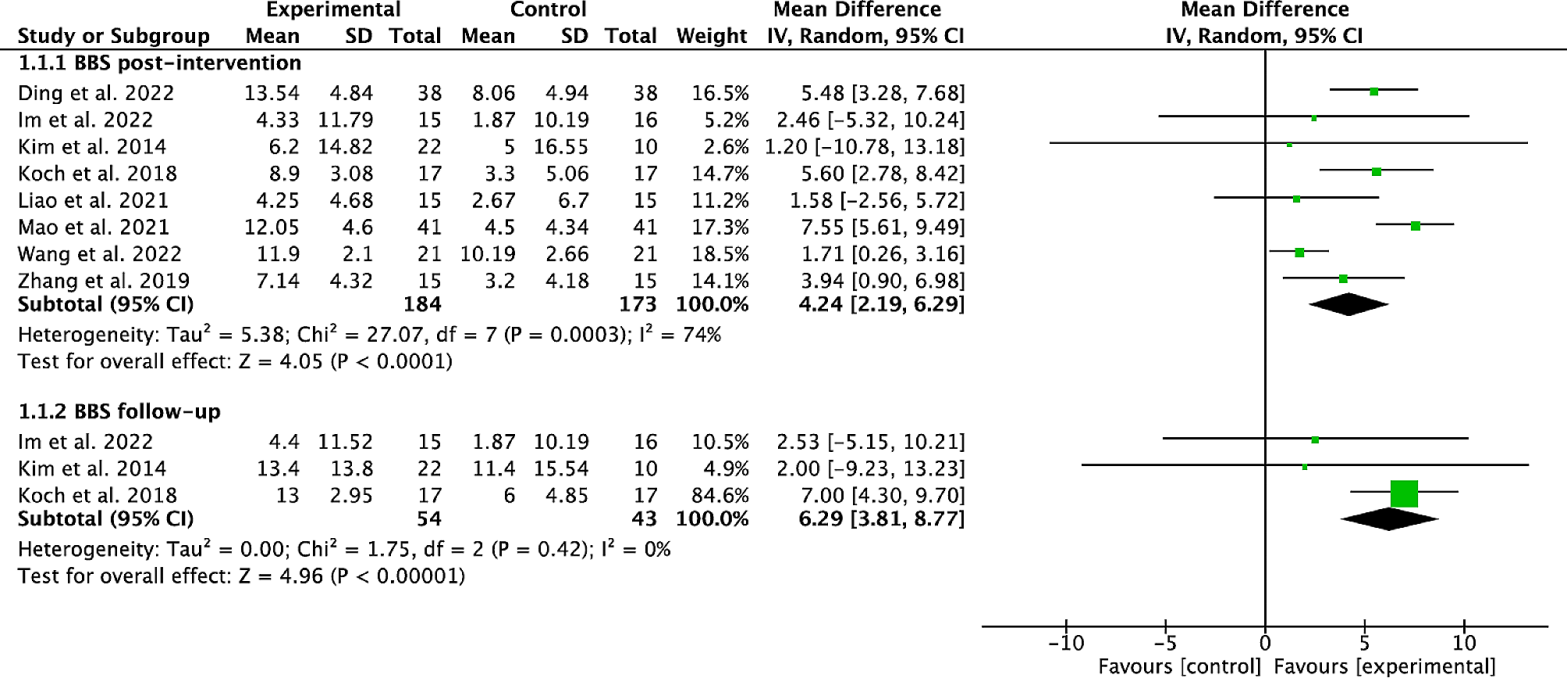
Forest plot of cerebellar TMS on BBS

Eight studies [21, 25, 26, 28, 30–33] with 357 stroke participants were pooled to estimate the overall effect of cerebellar TMS on BBS score. The pooled results from 8 studies with 357 participants showed that cerebellar TMS could significantly improve the post-intervention BBS score (MD = 4.24, 95%CI = 2.19 to 6.29, *P* < 0.00001; heterogeneity, *I*^*2*^ = 74%, *P* = 0.0003; Fig. 2).

The subgroup analysis showed that both subacute stroke participants (MD = 4.34, 95%CI = 2.68 to 6, *P* < 0.00001; heterogeneity, *I*^*2*^ = 2%, *P* = 0.38; Table 2) and chronic stroke participants (MD = 3.28, 95%CI = 0.22 to 6.35, *P* < 0.00001; heterogeneity, *I*^*2*^ = 65%, *P* = 0.06; Table 2) achieved significant post-intervention BBS improvement in experimental group. High-frequency TMS protocols (iTBS, rTMS with 10 HZ) induced significant post-intervention BBS improvement (MD = 4.42, 95%CI = 2.19 to 6.64, *P* = 0.0001; heterogeneity, *I*^*2*^ = 81%, *P* < 0.0001; Table 2) while low-frequency TMS protocols (cTBS, rTMS with 1 HZ) did not. Both small TMS sessions (≤ 10 sessions) (MD = 2.98, 95%CI = 0.69 to 5.28, *P* = 0.01; heterogeneity, *I*^*2*^ = 0%, *P* = 0.82; Table 2) and more TMS sessions (> 10 sessions) (MD = 5.02, 95%CI = 2.1 to 7.94, *P* = 0.0007; heterogeneity, *I*^*2*^ = 88%, *P* < 0.0001; Table 2) achieved significant post-intervention BBS improvement. Both ≤ 4500 pulse/week TMS protocols (MD = 1.71, 95%CI = 0.37 to 3.05, *P* = 0.01; heterogeneity, *I*^*2*^ = 0%, *P* = 1; Table 2) and > 4500 pulse/week TMS protocols (MD = 6.05, 95%CI = 4.86 to 7.24, *P* < 0.00001; heterogeneity, *I*^*2*^ = 33%, *P* = 0.21; Table 2) achieved significant post-intervention BBS improvement.

Both rTMS (MD = 5.67, 95%CI = 3.9 to 7.44, *P* < 0.00001; heterogeneity, *I*^*2*^ = 32%, *P* = 0.21; Table 2) and TBS (MD = 2.95, 95%CI = 0.29 to 5.61, *P* = 0.03; heterogeneity, *I*^*2*^ = 67%, *P* = 0.05; Table 2) achieved significant post-intervention BBS improvement.

The pooled results from 3 studies [21, 25, 26] with 97 participants showed that cerebellar TMS could significantly improve the BBS score at the end of follow-up (MD = 6.29, 95%CI = 3.81 to 8.77, *P* < 0.00001; heterogeneity, *I*^*2*^ = 0%, *P* = 0.42; Fig. 2). The subgroup analysis of BBS score at the end of follow-up were presented in Table 2.

### Effects of cerebellar TMS on TUG

**Fig. 3:**
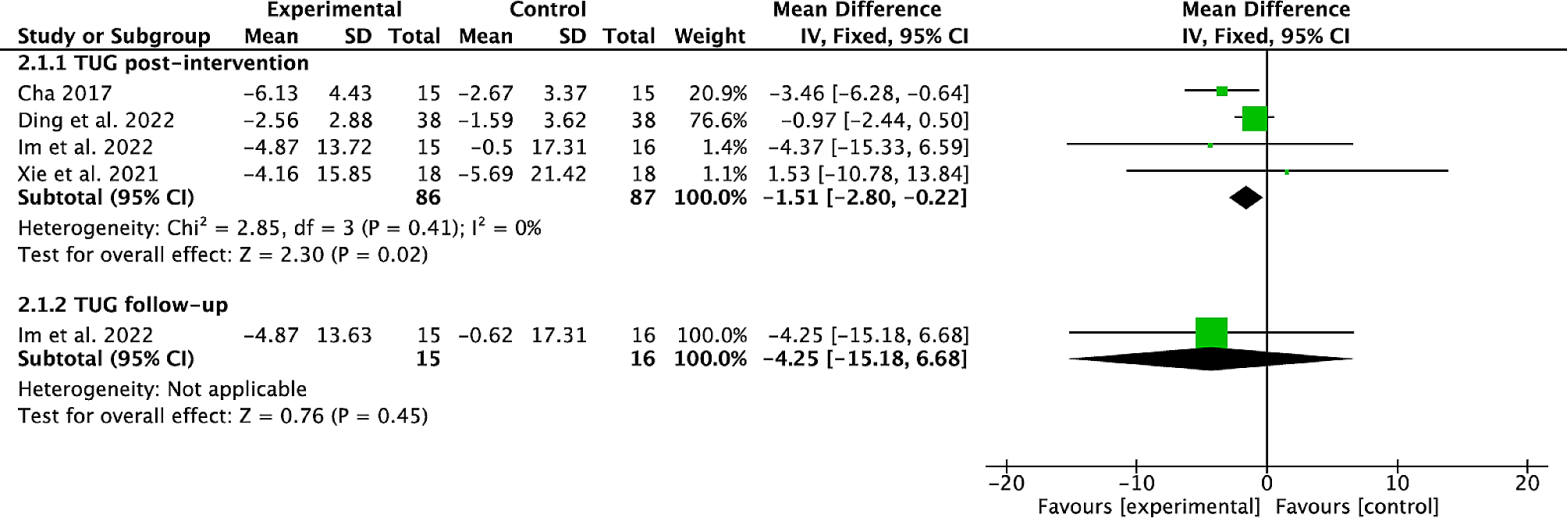
Forest plot of cerebellar TMS on TUG

Four studies [21, 24, 27, 31] with 173 stroke participants were pooled to estimate the overall effect of cerebellar TMS on TUG. The pooled results from 4 studies with 173 participants showed that cerebellar TMS could significantly improve the post-intervention TUG (MD=-1.51, 95%CI=-2.8 to -0.22, *P* = 0.02; heterogeneity, *I*^*2*^ = 0%, *P* = 0.41; Fig. 3).

The subgroup analysis showed that subacute stroke participants (MD=-1.47, 95%CI=-2.77 to -0.17, *P* = 0.03; heterogeneity, *I*^*2*^ = 23%, *P* = 0.27; Table 2), low-frequency TMS protocols (MD=-3.52, 95%CI=-6.24 to -0.79, *P* = 0.01; heterogeneity, *I*^*2*^ = 0%, *P* = 0.87; Table 2), > 10 sessions TMS protocol (MD=-1.5, 95%CI=-2.81 to -0.2, *P* = 0.02; heterogeneity, *I*^*2*^ = 58%, *P* = 0.12; Table 2), ≤ 4500 pulse/week TMS protocols (MD=-3.28, 95%CI=-5.94 to -0.62, *P* = 0.02; heterogeneity, *I*^*2*^ = 0%, *P* = 0.73; Table 2), rTMS (MD=-1.54, 95%CI=-2.84 to -0.25, *P* = 0.02; heterogeneity, *I*^*2*^ = 24%, *P* = 0.27; Table 2) achieved significant post-intervention TUG.

Only 1 study [21] reported the effect of cerebellar TMS on TUG at the end of follow-up, with no significant improvement (Fig. 3).

### Effects of cerebellar TMS on ADL

**Fig. 4:**
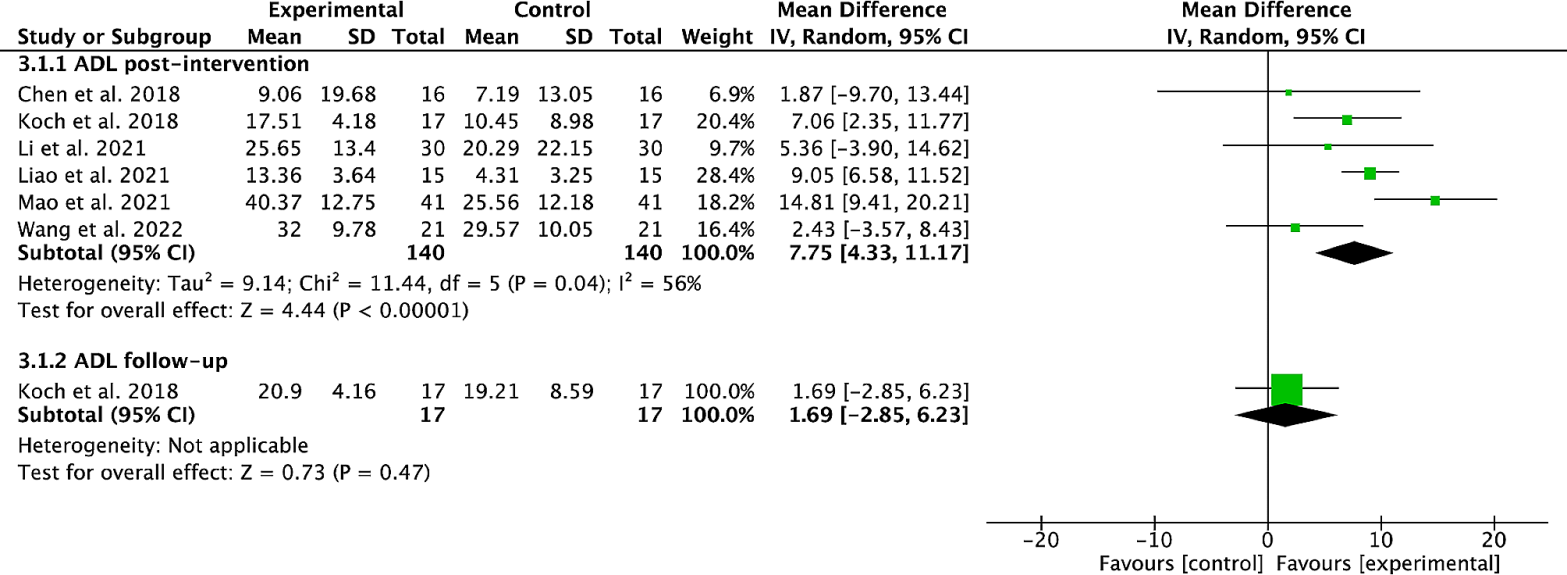
Forest plot of cerebellar TMS on ADL

Six studies [20, 22, 26, 28, 30, 33] with 280 stroke participants were pooled to estimate the overall effect of cerebellar TMS on ADL. The pooled results from 6 studies with 280 participants showed that cerebellar TMS could significantly improve the post-intervention ADL (MD = 7.75, 95%CI = 4.33 to 11.17, *P* < 0.00001; heterogeneity, *I*^*2*^ = 56%, *P* = 0.04; Fig. 4).

The subgroup analysis showed that both subacute stroke participants (MD = 8.53, 95%CI = 6.19 to 10.86, *P* < 0.00001; heterogeneity, *I*^*2*^ = 0%, *P* = 0.39; Table 2) and chronic stroke participants (MD = 5.29, 95%CI = 1.59 to 9, *P* = 0.005; heterogeneity, *I*^*2*^ = 29%, *P* = 0.23; Table 2) achieved significant post-intervention ADL improvement in experimental group. High-frequency TMS protocols induced significant post-intervention ADL improvement (MD = 7.95, 95%CI = 4.15 to 11.75, *P* < 0.00001; heterogeneity, *I*^*2*^ = 64%, *P* = 0.03; Table 2). Both small TMS sessions (≤ 10 sessions) (MD = 7.78, 95%CI = 2.4 to 13.15, *P* = 0.005; heterogeneity, *I*^*2*^ = 29%, *P* = 0.23; Table 2) and more TMS sessions (> 10 sessions) (MD = 7.68, 95%CI = 2.18 to 13.18, *P* = 0.006; heterogeneity, *I*^*2*^ = 70%, *P* = 0.02; Table 2) achieved significant post-intervention ADL improvement. Both ≤ 4500 pulse/week TMS protocols (MD = 5.71, 95%CI = 0.32 to 11.1, *P* = 0.04; heterogeneity, *I*^*2*^ = 61%, *P* = 0.08; Table 2) and > 4500 pulse/week TMS protocols (MD = 9.54, 95%CI = 3.66 to 15.42, *P* = 0.001; heterogeneity, *I*^*2*^ = 64%, *P* = 0.06; Table 2) achieved significant post-intervention ADL improvement.

TBS also achieved significant post-intervention ADL improvement (MD = 7.6, 95%CI = 5.63 to 9.58, *P* < 0.00001; heterogeneity, *I*^*2*^ = 26%, *P* = 0.25; Table 2).

Only 1 study [26] reported the effect of cerebellar TMS on ADL at the end of follow-up, with no significant improvement (Fig. 4).

### Effects of cerebellar TMS on other balance capacity outcomes

The pooled results from 3 studies [21, 24, 25] with 99 participants showed that cerebellar TMS did not significantly improve the post-intervention 10MWT comfortable walking time (Table 2). The pooled results from 2 studies [21, 25] with 63 participants showed that cerebellar TMS did not significantly improve the 10MWT comfortable walking time at the end of follow-up. Only 1 study separately reported that cerebellar TMS did not significantly improve the 10MWT (steps) post-intervention and at the end of follow-up [25], 10MWT (maximum walking time) post-intervention [25], ABC post-intervention and at the end of follow-up [21], and postural sway post-intervention [27].

The pooled results from 2 studies [32, 33] with 112 participants showed that cerebellar TMS significantly improved the SI (eyes open) post-intervention (MD=-4.47, 95%CI=-5.8 to -3.14, *P* < 0.00001; heterogeneity, *I*^*2*^ = 0%, *P* = 0.79; Table 2). The pooled results from 3 studies [31–33] with 188 participants showed that cerebellar TMS significantly improved the SI (eyes close) post-intervention (MD=-4.37, 95%CI=-5.58 to -3.15, *P* < 0.00001; heterogeneity, *I*^*2*^ = 0%, *P* = 0.65; Table 2). Only 1 study [29] reported that cerebellar TMS significantly improved the FMA-Balance post-intervention (Table 2).

### Methodological quality assessment of included studies

The PEDro scores were given between 5 and 10, with a mean score of 7.2. There were 12 studies ranging from 6 to 9, which indicating high-quality studies. Only 1 study with a score of 5, indicating a moderate methodological quality. The detailed PEDro scores for 13 included studies were displayed in Table 3.

### Sensitivity analysis

The leave-one-out approach was used for sensitivity analysis. The result of sensitivity analysis showed that the pooled effect were consistent for BBS post-intervention and at the end of follow-up, and ADL post-intervention. However, the effect of cerebellar TMS on TUG post-intervention become not significant after removing the study of Cha [27]. The heterogeneity changed to be low (heterogeneity, *I*^*2*^ = 39%, *P* = 0.13) for BBS post-intervention after removing the study of Wang et al. [30].

### Adverse events and side effects

There were 3 study reported the adverse events. One participants in the experimental group reported a mild headache at a level of 2/10, and did not require treatment to resolve the headache in the study conducted by Liao et al [28]. One participant complained of vertigo and discontinued treatment in the study conducted by Im et al [21]. One participant experienced transient mild headache during the TMS intervention, which self-relieved a few seconds later [29].

**Table 3:** PEDro score for Methodological Quality assessment of including studies

Reference	Item 1	Item 2	Item 3	Item 4	Item 5	Item 6	Item 7	Item 8	Item 9	Item 10	Item 11	Score
Xie et al. 2021	1	1	1	1	1	0	1	1	1	1	1	9
Liao et al. 2021	1	1	1	1	1	0	1	0	1	1	1	8
Li et al. 2021	1	1	0	1	1	0	0	1	1	1	1	7
Koch et al. 2018	1	1	1	1	1	0	1	1	0	1	1	8
Kim et al. 2014	1	1	1	1	1	0	1	0	1	1	1	8
Im et al. 2022	1	1	1	1	1	0	1	1	0	1	1	8
Chen et al. 2018	1	1	1	1	1	0	1	1	1	1	1	9
Cha 2017	1	1	0	1	1	0	0	0	0	1	1	5
Wang et al. 2022	1	1	0	1	1	0	1	1	1	1	1	8
Zhang et al. 2019	1	1	0	1	0	0	0	1	1	1	1	6
Mao et al. 2021	1	1	0	1	0	0	0	1	1	1	1	6
Ding et al. 2022	1	1	0	1	0	0	0	1	1	1	1	6
Duan et al. 2020	1	1	0	1	0	0	0	1	1	1	1	6

Note: Item 1 = eligibility criteria, Item 2 = random allocation, Item 3 = concealed allocation, Item 4 = similar baseline, Item 5 = subjected blinded, Item 6 = therapists blinded, Item 7 = assessors blinded, Item 8 = < 15% dropouts, Item 9 = intention-to-treat analysis, Item 10 = between-group comparison, Item 11 = point measures and variability data, 1 = described explicitly and in details, 0 = unclear, inadequately described

## Discussion

This systematic review and meta-analysis aimed to analyze the overall effect of cerebellar TMS (such as LF-rTMS, HF-rTMS, iTBS, and cTBS) on balance capacity and ADL for stroke patients. We also examined the influence of different stroke stages and TMS protocol parameters (i.e., TMS stimulation frequency, TMS sessions, total TMS stimulation pulses per week, TMS modes) on balance capacity and ADL. Overall, our study showed that the cerebellar TMS significantly improved the balance capacity (BBS, and TUG) and ADL of stroke patients.

Consistent with our findings, Wu et al.’s meta-analysis [34] demonstrated that noninvasive cerebellar stimulation improved BBS score of stroke patients from 4 studies in which 3 studies used TMS and 1 study used transcranial direct current stimulation (TDCS). However, Wu et al.’s meta-analysis [34] concluded that noninvasive cerebellar stimulation did not improve ADL of stroke patients from 2 studies, which is inconsistent with our results due to limited included studies and different cerebellar stimulation methods. In Wu et al.’s meta-analysis [34], literature search was conducted up to October 1, 2021. Additional new studies have been published in the past two years which were included in this meta-analysis. Xia et al.’s review also showed that cerebellar rTMS improved the spasticity, balance function and gait in stroke patients [18].

This meta-analysis have revealed that cerebellar TMS exhibited notable improvement in post-intervention BBS, TUG, and ADL for stroke patients. Regarding the stage of stroke, participants were subacute stroke in 9 included studies, chronic stroke in 3 included studies, and 1 study did not report the details of stroke stage. Our results showed that both subacute and chronic stroke patients had significant improvement in BBS post-intervention and ADL post-intervention. The pooled results of 3 studies found that cerebellar TMS could improve the TUG post-intervention in subacute stroke patients. We speculate that cerebellar TMS was suitable for various stage stroke patients to improve balance capacity and ADL. Regarding the TMS frequency, our results showed that cerebellar HF_TMS had significant improvement in BBS and ADL post-intervention. The pooled results of 2 studies found that cerebellar LF_TMS could improve TUG post-intervention. It seemed that cerebellar HF_TMS was more effective for the balance capacity and ADL in stroke patients. Regarding the TMS sessions, our results showed that more than 10 sessions could significantly improve the BBS, TUG post-intervention, and ADL post-intervention. At least 10 sessions of cerebellar TMS were required to significantly improve the balance capacity and ADL for stroke patients.

Regarding the TMS pulses per week, our results showed that both ≤ 4500 pulse/week and > 4500 pulse/week could improve BBS post-intervention and ADL post-intervention. Combined with the characteristics of included studies in this meta-analysis, at least 3000 cerebellar TMS pulse/week were needed to improve the balance capacity and ADL for stroke patients. Regarding the TMS modes (rTMS and TBS), our results showed that both cerebellar rTMS and TBS could improve BBS post-intervention and ADL post-intervention. Therefore, cerebellar TMS was beneficial for the balance capacity and ADL of stroke patients regardless of TMS modes. The sensitivity analysis showed that our results were stable for BBS and ADL post-intervention. After removing Cha’s study, the effect of cerebellar TMS on TUG post-intervention become not significant. The reason may be that Cha’s study adopted the longest cerebellar TMS duration (4 weeks), induced better TUG post-intervention compared with other 3 included studies. Additionally, subgroup analysis for the effect of cerebellar TMS on TUG post-intervention based on PEDro score showed that the reason maybe the relatively low methodological quality of Cha’s study. Future research should focus on enhancing the methodological quality of the research. After removing the study of Wang et al.’s study, the heterogeneity changed to be low for BBS post-intervention. The reason may be the stroke duration of participants in Wang et al.’s study was much more longer than other included studies. Additionally, the intervention protocol of experiment group vs. control group were iTBS combined with routine rehabilitation program vs. routine rehabilitation program in Wang et al.’s study, while the intervention protocol of experiment group vs. control group were iTBS (or rTMS) combined with routine rehabilitation program vs. sham iTBS (or sham rTBS) combined with routine rehabilitation program in the other included studies.

The Minimum Clinically Important Difference (MCID) is defined as the smallest change in a subjective outcome measure that confers a perceptible clinical benefit, aligning with the expectations of patients and healthcare providers [35]. The MCID values for BBS [36], TUG [37], and ADL [38] were defined as 5.9 points, 3.7 s, and 5.9 points. The finding of this meta-analysis showed that cerebellar TMS could significantly improve the post-intervention BBS score (MD = 4.24), and post-intervention ADL (MD = 7.75). Improvements in both BBS and ADL scores exceeded their respective MCID, indicating that cerebellar TBS has significant clinical relevance in enhancing the recovery of stroke patients. These findings suggest that cerebellar TBS is a promising therapeutic approach that merits wider adoption and application in clinical practice.

The cerebellum plays a pivotal role in the execution of movement and motor control [39]. Cerebellar TMS may improve motor symptoms in stroke patients, as evidenced by enhancements in scores on the BBS and ADL, through modulating cerebral motor cortical excitability and altering Purkinje cells activity [40, 41]. Purkinje cells within the cerebellar cortex exert inhibitory effects on the dentate nucleus, which, in turn, governs the motor cortex through the ventrolateral motor thalamus [42]. Consequently, cerebellar brain inhibition (CBI) denotes the suppression of the motor cortex induced by the activation of Purkinje cells [42]. Observations have illuminated that cerebellar stimulation possesses the capability to modulate CBI by inducing alterations in Purkinje cell activity [43]. This, in turn, leads to a sustained and polarity-dependent bidirectional adjustment of cerebellar excitability [43]. Cerebellar output influenced many brain areas, such as M1, premotor, prefrontal and parietal areas like the PPC [44], which are integral for the execution of daily activities and balance function. Cerebellar TMS may impinge on the specific set of interneurons dependent on aminobutyric acid–ergic activity [40], which plays an important role in brain plasticity during poststroke recovery [45]. It modulates cortical excitability of distant interconnected cortical areas by acting through common temporal, spatial and frequency domains [46], which could result in improved BBS and ADL scores. Previous study confirmed changes in corticomotor excitability due to the changes in cerebello-cerebral inhibition after low-frequency cerebellar rTMS was applied to healthy people [47]. The effect of cerebellar TMS may be associated with the cerebello-thalamocortical circuit. This association arises from the cerebellum’s capacity to modulate diverse motor functions by influencing the primary motor cortex (M1) and the corticospinal output pathways through the cerebello-thalamocortical circuit [48]. The cerebellar iTBS could promote long-term potentiation at the cerebellar cortex level with an effect on the interconnected posterior parietal cortex (PPC) of the contralateral lesioned hemisphere [49, 50]. The cerebellar iTBS also induced long-term potentiation to reinforce the cerebello-thalamo-cortical interactions cycling, which was crucial for spatial-motor learning and could be reflected in the BBS and ADL scores [26].

In the study by Liao et al. [28], the observed MEP amplitude over the affected hemisphere was notably reduced in the treatment group compared to the sham stimulation group following two weeks of cerebellar iTBS. This suggests a suppression of corticospinal excitability in the affected hemisphere. Additionally, Liao et al. [28] found that two weeks of cerebellar exhibited significant trunk impairment scale (TIS) scores increase compared with control group. Stroke patients frequently present postural control dysfunction, and better trunk performance was usually associated with better balance function [51]. Previous study also showed that trunk function were more related to balance capacity and ADL than extremity functions for stroke patients [52]. In the study by Koch et al. [20], three weeks of cerebellar iTBS could promote gait and balance recovery in patients with stroke by acting on cerebello-cortical plasticity. What’s more, cerebellar iTBS also decrease the step width which was considered a sign of gait stability improvement [20]. Low-frequency cerebellar rTMS on posterior circulation stroke subjects induced cerebellar excitability depression, enhancing the locomotor adaptative learning during routine physical therapy, leading to better motor function improvement [25]. Chen et al.’s study showed that 2 week cerebellar iTBS with conventional physical therapy decreased the Modified Ashworth Scale (MAS) score and Modified Tardieu Scale (MTS) score of affected elbow flexors and wrist flexors compared with sham stimulation, and the changes reached a clinical significance [22]. The cerebellar iTBS could also significantly decrease the average shear wave velocity (SMV) value of biceps brachii and flexor carpi radialis compared with sham stimulation [22]. Due to the improvement in upper limb muscle spasticity and increased range of motion, stroke patients could reduce limitations in their daily life activities, leading to an enhancement in their quality of life. Cha’s study showed that 4 week cerebellar low-frequency rTMS significantly improve the Wisconsin gait scale score compared with sham stimulation [27]. The improvement of gait was closely related to the improvement of hand function and ADL in stroke patients [53].

We acknowledge several limitation in our study. Firstly, this study included 13 studies, different studies have different TMS protocol details, including TMS modes, TMS frequency, TMS sessions, TMS pulses, although the stimulation site was all in the cerebellum. Those confounding factors made it difficult to identify the most effective cerebellar TMS protocol despite some subgroup analysis were already conducted. Secondly, the TMS period was relatively short (less than 4 weeks) in all included studies, and most included studies did not perform follow-up assessment, the lasting effect of cerebellar TMS on balance capacity and ADL in stroke patients could not be fully observed. Thirdly, the sample size of most included studies was relatively small, and the methodological quality of some studies need to be improved. Finally, it was not appropriate to conduct a funnel plot to evaluate the publication bias due to the limited number of studies included in each specific outcome.

Publication bias is a critical issue in systematic reviews and meta-analyses, as it can lead to an overestimation of the treatment effect and misinterpretation of the evidence. When drawing conclusions, include a note of caution regarding the potential influence of publication bias and its implications for the interpretation of the results. Future studies should be conducted with larger sample size and follow the consolidated standards of reporting trials statement to achieve higher quality. The sparse and mixed follow-up data might affect the interpretation of the long-term efficacy of cerebellar TMS. Future studies should improve the collection and reporting of follow-up data, which could include longer follow-up periods and standardized reporting of outcomes. In addition, only 3 of the included studies in this meta-analysis reported mild adverse effects. To bolster the evidence for the efficacy and safety of cerebellar TBS for stroke, detailed reporting of all adverse effects in future studies is advised. This should include a thorough description of adverse effect symptoms, duration, and how they were alleviated. Future studies could also explore the different effect of TMS in the cerebellum and other brain regions for stroke patients.

## Conclusion

This systematic review and meta-analysis provides up-to-date evidence into the effect of cerebellar TMS on balance capacity and ADL for stroke patients. The results demonstrated that cerebellar TMS could improve the balance capacity and ADL for stroke patients. The cerebellar TMS appeared to be a promising and safe option, with certain clinical implications on balance capacity and ADL for stroke patients. Furthermore, larger-sample, higher-quality, and longer follow-up randomized control trials are needed to explore the more reliable evidence of cerebellar TMS protocol in the balance capacity and ADL, and clarify potential mechanisms.

### Electronic supplementary material

Below is the link to the electronic supplementary material.

Supplementary Material 1

## Data Availability

No datasets were generated or analysed during the current study.
