# Supplementary material for: Cerebellar transcranial magnetic stimulation for improving balance capacity and activity of daily living in stroke patients: a systematic review and meta-analysis
Source: BMC Neurol. 2024 Jun 15;24:205. doi: 10.1186/s12883-024-03720-1 (PMC11179288; doi:10.1186/s12883-024-03720-1)
Supplement: Supplementary file 1 — Supplementary Material 1 [file 12883_2024_3720_MOESM1_ESM.docx]

Supplement Table 1. Search strategy in Pubmed.

| #1 | "Transcranial Magnetic Stimulation"[MeSH Terms] OR "magnetic stimulation transcranial"[Title/Abstract] OR "stimulation transcranial magnetic"[Title/Abstract] OR "Transcranial Magnetic Stimulations"[Title/Abstract] OR "theta burst stimulation"[Title/Abstract] OR "iTBS"[Title/Abstract] OR "cTBS"[Title/Abstract] OR "TMS"[Title/Abstract] OR "rTMS"[Title/Abstract] |
| --- | --- |
| #2 | "Stroke"[MeSH Terms] OR "Stroke"[Title/Abstract] OR "cerebrovascular accident*"[Title/Abstract] OR "cva"[Title/Abstract] OR "cerebrovascular apoplexy"[Title/Abstract] OR "brain vascular accident*"[Title/Abstract] OR "cerebrovascular stroke*"[Title/Abstract] OR "cerebral stroke*"[Title/Abstract] OR "cerebrovascular accident*"[Title/Abstract] |
| #3 | "cerebellum"[MeSH Terms] OR "cerebellum*"[Title/Abstract] OR "Corpus Cerebelli"[Title/Abstract] OR "cerebellar"[Title/Abstract] OR "parencephalon*"[Title/Abstract] |
| #4 | #1 AND #2 AND #3 |
